# Supplementary material for: ‘We have a plan for that’: a qualitative study of health system resilience through the perspective of health workers managing antenatal and childbirth services during floods in Cambodia
Source: BMJ Open. 2022 Jan 3;12(1):e054145. doi: 10.1136/bmjopen-2021-054145 (PMC8724583; doi:10.1136/bmjopen-2021-054145)
Supplement: Supplementary data [file bmjopen-2021-054145supp001.pdf]

**Supplement 1.** Interview guide in English for semi-structured interviews with health facility providers or health department staff.

**Flood experiences**

*Give example of recent flood in the area and confirm event (when, where, length of flood).* Can you tell me about the last flood that happened near your [facility/catchment area]?

> *Explore*: Effect on villages and health

Can you describe your experiences of working during a flood?

> *Explore:* Changes/differences in care compared to no floods

**Provision and maintenance of services**

When there is a flood, what happens to prenatal care services at your [facility/catchment area]? What happens to delivery care services?

> *Explore:* Demand and access to care, staff changes, supplies, medicines, user fees, budget, management from upper levels

Can you describe a time when a woman was not able to get prenatal care at a [facility] during a flood? What happened? What about a time when a woman was not able to get delivery care at a [facility]? What happened then?

**Anticipating and coping with uncertainty**

How do you prepare prenatal services for the rainy season? How do you prepare delivery services?

Can you share specific examples of things that you do at the [facility/health department] to make sure that pregnant women are able to continue getting care during floods?

**External factors influencing the health system**

What are some reasons that your [facility/health department] might not be able to provide prenatal care during floods? What about for delivery care?

> *Explore:* Support and work with other departments/sectors/NGOs/committees, transport and access to facilities, supply chain, available funds, staff personal lives and priorities, changes in health, emergencies

What do you think influences pregnant women to come to your facility for prenatal care during floods? What about for delivery care?

**Interaction with the community**

How does the [facility/health department] work with pregnant women in the village during floods?

> *Explore:* Reasons for visiting other providers or home delivery, outreach in villages, input and accountability with community

In your opinion, how do you think the pregnant women feel about the prenatal care that they can get at [facilities] during floods? What about delivery care?

> *Explore:* Trust and quality, social media, feeling of ownership

**Gathering and using knowledge**

What kind of decisions do you have to make about services for prenatal care during floods? For delivery care?

> *Explore:* Referring patients to hospital, emergency obstetric cases, sources of information, communication with other departments/facilities/committees, flexibility and ability to make decisions

From your experience working during floods, what have you learned about providing prenatal care during floods? What have you learned about providing delivery care?

Can you give me an example of something you would like to know when there is a flood that would help make prenatal care better during floods? And for delivery care?
