# Supplementary material for: ‘We have a plan for that’: a qualitative study of health system resilience through the perspective of health workers managing antenatal and childbirth services during floods in Cambodia
Source: BMJ Open. 2022 Jan 3;12(1):e054145. doi: 10.1136/bmjopen-2021-054145 (PMC8724583; doi:10.1136/bmjopen-2021-054145)
Supplement: Author's manuscript [file bmjopen-2021-054145.draft_revisions.pdf]

BMJ Open is committed to open peer review. As part of this commitment we make the peer review history of every article we publish publicly available.

When an article is published we post the peer reviewers' comments and the authors' responses online. We also post the versions of the paper that were used during peer review. These are the versions that the peer review comments apply to.

The versions of the paper that follow are the versions that were submitted during the peer review process. They are not the versions of record or the final published versions. They should not be cited or distributed as the published version of this manuscript.

BMJ Open is an open access journal and the full, final, typeset and author-corrected version of record of the manuscript is available on our site with no access controls, subscription charges or pay-per-view fees (http://bmjopen.bmj.com).

If you have any questions on BMJ Open's open peer review process please email info.bmjopen@bmj.com

BMJ Open

‘We have a plan for that’: A qualitative study of health systems resilience through the perspective of health workers managing antenatal and childbirth services during floods in Cambodia

Journal:	*BMJ Open*
Manuscript ID	bmjopen-2021-054145
Article Type:	Original research
Date Submitted by the Author:	08-Jun-2021
Complete List of Authors:	Saulnier, Dell; Karolinska Institutet, Department of Global Public Health<br>Thol, Dawin; National Institute for Mental Health<br>Por, Ir; National Institute of Public Health<br>Hanson, Claudia; Karolinska Institutet, Department of Global Public Health; London School of Hygiene & Tropical Medicine<br>Von Schreeb, Johan ; Karolinska Institutet, Department of Global Public Health<br>Alvesson, Helle; Karolinska Institutet, Department of Global Public Health
Keywords:	HEALTH SERVICES ADMINISTRATION & MANAGEMENT, Change management < HEALTH SERVICES ADMINISTRATION & MANAGEMENT, QUALITATIVE RESEARCH, PUBLIC HEALTH

SCHOLARONE™
Manuscripts

I, the Submitting Author has the right to grant and does grant on behalf of all authors of the Work (as defined in the below author licence), an exclusive licence and/or a non-exclusive licence for contributions from authors who are: i) UK Crown employees; ii) where BMJ has agreed a CC-BY licence shall apply, and/or iii) in accordance with the terms applicable for US Federal Government officers or employees acting as part of their official duties; on a worldwide, perpetual, irrevocable, royalty-free basis to BMJ Publishing Group Ltd ("BMJ") its licensees and where the relevant Journal is co-owned by BMJ to the co-owners of the Journal, to publish the Work in this journal and any other BMJ products and to exploit all rights, as set out in our licence.

The Submitting Author accepts and understands that any supply made under these terms is made by BMJ to the Submitting Author unless you are acting as an employee on behalf of your employer or a postgraduate student of an affiliated institution which is paying any applicable article publishing charge ("APC") for Open Access articles. Where the Submitting Author wishes to make the Work available on an Open Access basis (and intends to pay the relevant APC), the terms of reuse of such Open Access shall be governed by a Creative Commons licence – details of these licences and which Creative Commons licence will apply to this Work are set out in our licence referred to above.

Other than as permitted in any relevant BMJ Author's Self Archiving Policies, I confirm this Work has not been accepted for publication elsewhere, is not being considered for publication elsewhere and does not duplicate material already published. I confirm all authors consent to publication of this Work and authorise the granting of this licence.

For peer review only

Title

‘We have a plan for that’: A qualitative study of health systems resilience through the perspective of health workers managing antenatal and childbirth services during floods in Cambodia

Authors

1. Dell D Saulnier, PhD – Department of Global Public Health, Karolinska Institutet, Stockholm, Sweden.

   Corresponding author address: Karolinska Institutet, Widerströmska huset, Plan 3, Tomtebodavägen 18a, 171 77 Stockholm, Sweden. *Phone*: +46 733 232 076. *Email:* dell.saulnier@ki.se

2. Thol Dawin, MD, MPH - National Institute of Public Health, Phnom Penh, Cambodia
3. Ir Por, MD, PhD - National Institute of Public Health, Phnom Penh, Cambodia
4. Claudia Hanson, MD, PhD - Department of Global Public Health, Karolinska Institutet; Department of Disease Control, London School of Hygiene and Tropical Medicine
5. Johan von Schreeb, MD, PhD - Department of Global Public Health, Karolinska Institutet
6. Helle Mölsted Alvesson, PhD - Department of Global Public Health, Karolinska Institutet

Keywords

Health systems resilience; floods; shocks; Cambodia; health service provision

Word count: 4 057

For peer review only

For peer review only - http://bmjopen.bmj.com/site/about/guidelines.xhtml

ABSTRACT (300 words)

Objective Health systems resilience has been promoted as one way to increase a system's ability to deal with shocks like floods. Studying health systems that currently exhibit the capacity for resilience when shocked could enhance our understanding about what generates and influences resilience. This study aimed to generate empirical knowledge on health systems resilience by exploring how public antenatal and childbirth health services in Cambodia have absorbed, adapted, or transformed in response to seasonal and occasional floods.

Design A qualitative study using semi-structured interviews and thematic analysis and informed by the Dimensions of Resilience Governance framework.

Setting Public sector healthcare facilities and health departments in two districts exposed to flooding

Participants Twenty-three public sector health professionals with experience providing or managing these services during recent flooding

Results The theme 'Collaboration across the system creates adaptability in the response' reflects how collaboration and social relationships among providers, staff, and the community have delineated boundaries for actions and decisions for antenatal and childbirth care during floods. The floods were perceived as having a modest impact on health services. Knowing the boundaries and having preparation and response plans let staff prepare and respond in a flexible yet stable way. The theme was derived from ideas of: i) seasonal floods as a minor strain on the system compared to persistent, system-wide organizational stresses, ii) the ability of the health services to adjust and adapt flood plans, iii) a shared purpose and working process during floods, iv) engagement at the local level to fulfill a professional duty to the community, and v) creating relationships between health system levels and the community to enable flood response.

Conclusion The capacity to absorb and adapt to floods was seen among the public sector services. Strategies that enhance stability and flexibility may foster the capacity for health systems resilience.

ARTICLE SUMMARY

For peer review only - http://bmjopen.bmj.com/site/about/guidelines.xhtml

Strengths and limitations of the study

- Participants in a variety of roles and from multiple levels of the health system strengthened the diversity of perspectives on health service functioning during floods.
- Extensive discussion among the diverse research team following all interviews and a comprehensive audit trail increased the dependability and credibility of the study.
- The data collectors were familiar with rural Cambodia, its health system, and health services at multiple levels, which helped the quality of the interviews.
- Participants at higher levels of authority may have provided more socially desirable information or refrained from sharing the full extent of their experiences or thoughts.
- The participants' rationale for their actions when preparing and responding to floods was not always articulated, which may affect the reliability of the results.

For peer review only - http://bmjopen.bmj.com/site/about/guidelines.xhtml

INTRODUCTION

Since early 2020, health systems worldwide have been severely challenged by the COVID-19 pandemic. Systems have faced a sudden demand to surveille, diagnose, and care for people with COVID-19 and the continued demand for essential services like vaccination and maternal healthcare;[1] in some cases, the pandemic has already disrupted these services.[2] Attention has been increasingly placed on health systems resilience as a way to reduce health system vulnerability and increase their ability to deal with shocks like the 2014-2016 West Africa Ebola outbreak, the COVID-19 pandemic, or extreme weather events.[3–5] Extreme weather events are projected to become more frequent and severe as a consequence of climate change, including an increase in seasonal floods and occasional floods.[6] By the end of the century, an additional 2.3 billion people are expected to be at risk of flooding.[7] The health impacts of seasonal and occasional floods are diverse, from direct mortality and morbidity to indirect poor health from their impact on societal functioning.[8,9] Health systems, crucial to managing the health of the population, are also directly and indirectly impacted by floods.[9–11]

Resilient health systems have the capacity to continue delivering health services when shocked or stressed, responding and adapting to the new health needs created by the shock or stress and the routine health needs of the population.[3,12,13] Shocks are commonly described as sudden or extreme external phenomena that impact healthcare demand and health system resources; stresses are chronic, internal strains on system functioning, like persistent underfunding.[14] The Dimensions of Resilience Governance framework suggests that if a health system is able to integrate and process *knowledge,* anticipate and cope with *uncertainty*, manage interactions with other systems at multiple levels (*interdependence*), and create a socially and contextually accepted system (*legitimacy*), it will be capable of managing shocks.[15] Health systems can then absorb shocks using existing strategies and resources, adapt to them by temporarily adjusting how resources are used, or transform the structure of the system in the long-term to avoid shocks.[5,16,17]

To date, the literature has focused on how to define and understand the concept of health systems resilience; less has been published on how resilience can be generated.[5,14,18] Lessons have been taken

For peer review only

from organizational resilience and from other complex adaptive systems challenged by shocks.[13,18,19] Exploring what makes health systems resilient in real-world situations is necessary to understand what capacities could foster resilience to shocks like floods.

Cambodia is regularly exposed to repeated seasonal and occasional floods, as well as flood disasters. Improving health service delivery readiness to operate during floods has been an ongoing priority since 2008,[20,21] and the health services have remained functional during previous flood disasters.[22] Concurrent with reforms that have focused on strengthening health service delivery and management, several strategic plans have been developed to enhance the health system's ability to respond to floods.[20,23,24] This study therefore aims to understand how health system resilience is generated, by exploring the capacity of the Cambodian health system to absorb, adapt, and transform during floods. Guided by the capacities in the Dimensions of Resilience Governance framework [15] we sought to understand how healthcare workers in public sector health facilities and health departments are able to continue providing health services during seasonal and occasional floods, using antenatal and childbirth services as indicators of routine and new health needs, respectively.[25,26]

METHODS

Study setting

In Cambodia, the National Committee for Disaster Management leads a national-to-district level program for disaster management and flood response.[27] Operational preparedness and response plans exist for multiple levels of the health system and include items such as roles and responsibilities during disasters (Box 1). During floods, public health facilities conduct additional health promotion, education, and clinical outreach, and health departments prepare resources and oversee the flood response. Routine antenatal and uncomplicated childbirth care is provided at public health centers and private clinics, per the patient's choice.[28] Additional antenatal services and emergency obstetric care is provided at private clinics and public hospitals.[29]

Study design, participants, and recruitment

For peer review only - http://bmjopen.bmj.com/site/about/guidelines.xhtml

This qualitative study used semi-structured interviews to explore public sector healthcare providers and health department staff experiences in delivering and managing antenatal health services during floods. Two districts were selected: one that typically floods every year (seasonal flooding) and one that experiences floods less often (occasional flooding) to cover the main frequencies of flooding in Cambodia.[30] Each district had flooded in the previous five months. A heterogeneous sample of participants who had been working in their current role during the most recent flood were purposively selected from five public health centers, two district referral hospitals, two provincial and two district health departments, two communes, and the Ministry of Health (Table 1).

Data collection

Two female Cambodian data collectors (a midwife studying public health and a qualitative researcher and retired medical school lecturer) conducted and audio recorded 23 semi-structured interviews in Khmer at the end of the rainy season in 2018. The topic guides used the four theoretical dimensions from the Dimensions of Resilience Governance framework [15] to investigate participants' real-life experiences working with service provision and management during floods (Supplement 1). Participants were interviewed individually in private areas such as empty meeting rooms. The second data collector had taught five of the participants in medical school since the 1980s; she acknowledged the connections and kept the interview focused on the topic guides. The quality of the interviews was assessed continually among the team through discussion and field notes using the information power concept and the 23 interviews captures the relevant information.[31] Interviews were transcribed verbatim into Khmer and translated into English.

Table 1. Characteristics of the study sites and participants

[Note: Removed by editor at acceptance. Please see final version of manuscript.]

Data analysis

A data-driven thematic analysis [32], conducted in Nvivo 12 Pro, was chosen to ensure that the theoretical concepts of the framework did not eclipse other relevant information in the data about the health system's functioning and to capture ideas that spanned multiple dimensions in the framework. Codes were

For peer review only - http://bmjopen.bmj.com/site/about/guidelines.xhtml

developed by DS that reflected descriptions or ideas about influences on health service delivery during floods. The relationship between codes was explores using the framework's concepts of knowledge, uncertainty, interdependence, and legitimacy. Categories were then created from observed patterns of meaning plus the theoretical understanding gained during the previous step and a single theme was identified. Lastly, the findings were mapped back onto the framework to illustrate how they contributed to the framework's capacities (Figure 1)

Patient and public involvement

No patients involved.

Ethical considerations

The National Ethics Committee for Human Research in Cambodia (ref: 204 and 276) and the Swedish Ethical Review Authority (Dnr: 2019-02458) approved the study. All participants gave written or verbal consent prior to the interviews. One participant requested that part of their interview be excluded from transcription and analysis.

RESULTS

We identified one theme, 'Collaboration across the system creates adaptability in the response', with five categories (Table 2). Collaboration and social relationships appeared to create clear boundaries to decision-making around antenatal and birth care during floods. Both seasonal and occasional floods were discussed as strains rather than shocks. With a firm understanding of the boundaries, providers and staff were able to prepare and respond to these floods in a flexible but stable manner, resulting in absorptive and adaptive capacity. Overarching health system and contextual changes appear to be fundamentally changing public sector service use and function during floods, potentially transforming the system and the impact of floods themselves.

Table 2. The theme, categories, and subcategories describing the influences on public sector antenatal and childbirth health services during floods

Theme	Categories	Sub-categories
Collaboration across	Floods just another strain on service delivery	

For peer review only - http://bmjopen.bmj.com/site/about/guidelines.xhtml

the system creates adaptability in the response	Facilities and health departments able to calibrate and maneuver to make flood routines work for them	
	Working in the same direction during floods	
	Engaging in local governance to fulfill a duty to the community during floods	Health centers becoming obsolete during floods<br>Believing in the value of the system during floods
	Creating relationships to successfully respond to floods	Health centers as the lynchpin for the health response to floods<br>Social relationships can challenge information sharing

‘Floods just another strain on services’

Participants did not perceive seasonal or occasional floods as a threat to health service functioning. Instead, they were part of a larger constellation of constant stresses to service delivery. Providers and health department staff in both districts described pregnancy, childbirth, and other health problems during floods as routine and “nothing serious”. They note that antenatal and childbirth services usually functioned as normal during both types of floods. Changes over time, like better roads, let participants feel that floods were no longer a major threat:

> *The floods are not so big that we have to focus on them so much. They’re not big to the point of an emergency.* (IDI 7, provincial health department)

Participants described numerous, persistent challenges to service delivery that were independent from flooding. They included i) slow, unreliable referral systems, ii) inadequate professional training, iii) heavy workloads and staff shortages, iv) unintegrated and undependable support from not-for-profit organizations, v) poor or inaccurate health and system performance information, and vi) a slow and unresponsive process for requesting funds, resources, and help from higher levels of the health system. Floods exacerbated these challenges but everyday strains on service provision subsumed the effect of floods.

‘Facilities and health departments able to calibrate and maneuver to make flood routines work for them’

For peer review only

For peer review only - http://bmjopen.bmj.com/site/about/guidelines.xhtml

Staff at facilities and departments reviewed the standard flood response plans each year. Together with flood warnings and their experiences from previous floods, participants in both districts discussed how they knew the routines for preparing. They began to actively prepare in the months and weeks preceding the expected floods:

> *All the plans are already written […] We have planned what to do, we have all events organized, and we just follow accordingly. We have written everything into the yearly plan, arranged it by month as well. And if there's a flood, we have listed down already what we need to do, what to buy, and so on.* (IDI 2, health center chief)

According to participants, facilities and health departments in both districts calibrated their plans and routines to fit different scenarios, and flexibly manoeuvred during flood response. They adapted the standardized plans—formulated for disasters—to fit large- and smaller-scale floods in each district. The adaptions were based on previous experience and scenarios they might encounter. The plans used strategies like: i) diverting clinical staff between facilities and health departments to cover shortages or supplement outreach teams, ii) using discretionary budgets to prepare or respond, iii) piggybacking on the non-health response to floods, and iv) choosing how to organize outreach for flood-related care:

> *We have a boat, it's even a motorboat. It costs more to transport the boat to the [flooded] area than it does to rent a local boat there. We ride a motorbike and then ride on the boat with the locals. It costs less.* (IDI 18, district health department)

'Working in the same direction during floods'

Facility chiefs and health department staff described a multilevel, multisectoral flood response in both districts. Bigger floods required a larger response and more organization at the provincial level with external sectors. Participants knew that they could not control problems outside the health sector that affected health or their work during floods and needed to trust other sectors and higher authorities to find solutions.

Participants noted that they had to collaborate between levels of the health system to keep facility-based services and clinical outreach functional during floods. They relied on higher levels to be responsible for

For peer review only - http://bmjopen.bmj.com/site/about/guidelines.xhtml

problem-solving or to help with supplies, staff, and expertise; were free and able to contact higher levels for help, and described a strong sense of teamwork across levels when responding to floods:

When there's a big flood, the OD will have to visit the flooded areas every day to see the people's situation, how they are living, is there any clean water for them to use, any diarrhea, are there any health center staff to help them with their health problems or not? Everyone is enthusiastic when there's a big flood, both the provincial level and district level. (IDI 10, district health department)

While free to ask for help, participants described clear limits to their decision-making. They stayed within the boundaries of their designated role when preparing and responding to floods. Their superiors made and approved most decisions about responding to bigger floods, noted in particular by midwives and health department staff:

The decisions that I can make during a disaster without asking the upper level to help are none. During a disaster, you have to depend on the upper level. I have to report to them because we cannot work alone. (IDI 9, provincial health department)

'Creating relationships to successfully respond to floods'

Health department staff and providers were able to share information and keep facility-based care and outreach services functioning during floods, facilitated by their relationships with each other and the community.

Health centers as the lynchpin for the health response to floods

Participants saw the existing relationships between health center staff and the community as crucial for providing health services during floods. Health center staff worked to keep outreach and facility-based services functioning smoothly during floods. For example, they would pay out-of-pocket for gasoline or reimburse village leaders for emergency phone calls. Health center staff felt that the community's knowledge of health risks and strategies to stay healthy during floods made it easier to provide services:

The health center doesn't have a boat, not even a tuk tuk [to transfer patients during floods]. The women need to have their own boats. The villagers are very smart. They know that they need to have a boat with them when they come for antenatal care. (IDI 14, health center midwife)

For peer review only - http://bmjopen.bmj.com/site/about/guidelines.xhtml

Health centers and departments needed villages to supply information on their health and the severity of the floods in their area so that they could respond appropriately. According to participants, health centers needed to negotiate with commune and village leaders to allow health department staff to enter to villages during floods:

> *Before we go to any village, we inform the health center to inform [the local authorities]. If you don't inform them, they won't know what you're doing and there will be trouble. If it's before an election, you would have to be even more careful.* (IDI 18, district health department)

Social relationships can challenge information sharing

Participants described flood and health information as accessible to staff across all levels of the health system, structured to flow up or down from the village to the national level. Contact networks were openly accessed, regardless of rank or role. Open communication and information sharing meant contact was often between individuals rather than roles:

> *Before they [the health center] transfers a case here, they will call us. They call the hospital, there's a telephone there. Sometimes they contact [the hospital chief] directly. Sometimes they call any staff that they know here.* (IDI 20, referral hospital midwife)

Personal and social relationships could hamper access and information sharing between individuals. Lower-ranking staff in health departments and facilities, like midwives, only received information about how their facility would respond to floods on a 'need-to-know' basis. Participants explained that they were more willing to contact known, trusted individuals for information or help. Without trust, participants felt obligated to follow up on information themselves to ensure it was correct:

> *Sometimes, the information that is available is not clear. Then we have to go to the community for an assessment. Like in the past, we had information from the National Committee for Disaster Management that there were deaths [during the flood] but then when I went there, they [the health department] said there were none. So it's hard.".* (IDI 23, Ministry of Health)

'Engaging in local governance to fulfill a duty to the community during floods'

For peer review only - http://bmjopen.bmj.com/site/about/guidelines.xhtml

Staff at the health centers remained engaged in holding themselves accountable for the care they delivered. They described feeling a professional duty and responsibility to the community's health during floods, despite feeling progressively less relevant in a changing landscape.

Health centers becoming obsolete during floods

Participants described health centers as struggling to compete for services with private facilities and public hospitals regardless of floods. Health center providers noted more women now chose facilities with better services and resources, and expressed difficulty understanding why they were losing relevance. Health center participants felt obligated to focus on facility-based services rather than outreach for flood-related care and felt tied to their facilities during floods. They had to prioritize staff, funds, and equipment for the health center, and felt less able to provide comprehensive antenatal outreach during floods:

> *We have only one electric generator that is used at the health center. So we cannot take that into the village [during outreach]. If we do, when people come to the health center for treatment, then we can't provide full services to them.* (IDI 4, health center midwife)

As a result, clinical outreach during floods began when community members could no longer reach the health centers and focused on preventing communicable diseases.

Believing in the value of the system during floods

During floods, health centers described attempts to increase demand at their facilities, using schemes like waiving services fees and promoting facility-based care. They actively attempted to improve service quality at their facilities, for instance by holding providers accountable for perceived poor treatment of patients.

Providers and health department staff felt pride in their work and a professional duty to serve the community, despite the persistent challenges described earlier. When faced with floods, they discussed sharing a common goal of helping the system work as it should, regardless of potential challenges. This echoed earlier expressions about relying on higher levels of the health system and the community to take responsibility:

For peer review only - http://bmjopen.bmj.com/site/about/guidelines.xhtml

Cases of [women] unable to deliver, we never have them. We don't have any issue with it because we have the network to help her on time. If there are any problems, the village and commune chiefs have my phone number and the district head's phone number, if the patient needs an ambulance to take her from home. (IDI 19, hospital chief)

Based on the findings above, we illustrate the relationship between the theme and the Dimensions of Resilience Governance framework in Figure 1. The four dimensions feed into the process outlined in the theme, showing how boundaries on decision-making are contributing to preparation and response to modest seasonal and occasional floods, helping to create the capacity to absorb, adapt, and transform.

Figure 1. A visual representation of the four dimensions of resilience and the system's capacity to absorb, adapt, and transform as they relate to the main theme, using health center outreach services for antenatal and childbirth care during a flood as an example

DISCUSSION

This qualitative study highlights the importance of understanding the practices that enable health service provision during flooding. The results show an adjustable approach to preparing and responding to floods. The approach is grounded in collaboration and relationships across the system that set boundaries around actions and decisions, giving the system the capacity to adapt and absorb floods. This approach was similar between the seasonal and occasionally flooded districts, despite the difference in flood frequency. Health system strengthening initiatives and developmental changes in Cambodia, like reforms to promote and improve facility-based maternal health services and better roads, have transformed the health system context over time. The boundaries around actions and decisions, coupled with the transformative changes, had led to a system that is capable of maintaining pregnancy and childbirth services during floods, and one that views floods as strains rather than shocks.

The absorptive and adaptive capacities were characterized by the health facilities' ability to prepare and respond to seasonal and occasional floods in a stable but flexible way, with support from the health departments. Transformative capacity was not readily reported by the participants. Some changes in the

For peer review only - http://bmjopen.bmj.com/site/about/guidelines.xhtml

structure or functioning of the system, such as the general shift to facility-based antenatal and childbirth care, were not explicitly linked to flooding. If the three capacities are seen as ‘different perspectives of the same reality’,[33] stability and flexibility are both necessary:[13,34] stability to cushion against shocks and let coordinated actions emerge, and flexibility to change and adapt. In our results, previous experience and planning created the stability to absorb and adapt, and the flexibility to continue adapting to the current flood if needed.

A driver of the stability and flexibility appeared to be the relationships between individuals and groups that delimited actions and decision-making for services during floods. Health systems are populated by health workers and community members with the power to make choices, and the social networks, relationships, and collaborations among them have been identified as influencers of resilience.[13,17,19,33,34] In this study, relationships influenced how knowledge was collected and shared during floods. Being able to rely on higher levels and other providers or staff was fundamental to the interdependent preparation and response described by the participants. Understanding the boundaries of the decision-making space appears as a key component to taking action, as seen in a study of decision-making in Uganda.[35] In the context of the Cambodian health system, where decision-making is already hierarchical,[36,37] top-down leadership may have helped counteract uncertainty when responding to floods; during floods, participants focused on actions and decisions within their own responsibilities, shifting decisions beyond their remit upwards in the hierarchy. However, further work would be needed to understand and compare the effects of hierarchy on uncertainty and decision-making in nonhierarchical contexts.

The descriptions by health center providers of their close, positive relationships with local community members contrast with their descriptions of a steady decrease in visits to health centers. An earlier study on community management of pregnancy and childbirth during floods identified low degree of trust and sense of ownership in health centers among community members.[38] In this study, participants described trying to increase trust and demand for services year-round, regardless of floods. This could be linked to their perception of floods as a strain to the system rather than a shock. The main challenge identified

For peer review only - http://bmjopen.bmj.com/site/about/guidelines.xhtml

during floods was not coping with or responding to health needs, but how the usual conditions of the public health system constrained the quality or quantity of care the public health system could give. Without a strong basis of trust and quality when floods were not present, there may have been fewer incentives for community members to visit public health centers during floods, when barriers to care were higher.[38] Still, the participants appeared to have a strongly anchored belief in their roles and the care they provide, and were able to build relationships with community members, which may be seeds for constructing a more legitimate system.[12,19,39,40]

Further differentiating the concepts of shocks and stresses could be useful to understand how characteristics of resilience might vary depending on what challenges a system faces.[13,41] Both seasonal and occasional floods were described as a strain on the health system—acute, external events that were also chronically repetitive in nature and aggravated existing everyday challenges—bridging current definitions of shocks and stresses.[14] This suggests that the normal pattern of floods in the districts are not a major challenge to the health services. Since floods are projected to gradually become more frequent with climate change, this is an argument for promoting the concept of everyday resilience towards repeated seasonal and occasional floods, that can in turn promote resilience to more acute, extreme shocks.[3,5]

Study limitations

Some desirability bias is likely present among participants at higher levels of authority, such as the health departments, when it was harder to achieve depth and capture informal views than with lower-level participants. Interviews with the participants who were previous students of a data collector were checked for respondent and researcher biases; there were no clear differences. Interviews that gave practical examples of participants' rationale for their actions during floods were considered higher quality in the study. Using a nascent conceptual framework with overlapping dimensions meant data had to be abstracted to a high degree during analysis. We dealt with this by using a data-driven approach during coding and then allowed the categories and themes to be influenced by the framework.

CONCLUSIONS

For peer review only - http://bmjopen.bmj.com/site/about/guidelines.xhtml

Public health facilities and health departments appeared to have the capacity to absorb and adapt to seasonal and occasional floods in Cambodia. The boundaries on decisions and actions and pre-existing flood plans for facilities and departments at multiple levels created stability and flexibility when preparing and responding to floods. The apparent success of the system in responding to floods leads us to conclude that strategies that enhance stability and flexibility may foster the capacity for health systems resilience. However, the impact of the floods in this study were minor compared to persistent, system-wide challenges to health service functioning. Health systems that are chronically strained by repeated external events may benefit most from health system strengthening efforts to everyday challenges that can pay off during stronger shocks.

For peer review only

For peer review only - http://bmjopen.bmj.com/site/about/guidelines.xhtml

ACKNOWLEDGEMENTS

The authors would like to thank all the clinicians and staff who gave their time to take part in this study. We also thank the data collectors for their outstanding work, Sophea Men for her translation of the transcripts, and Vannarath Te for his contributions during piloting.

FUNDING STATEMENT

This work was supported by the Swedish Research Council (Dnr: 2016-05678)

COMPETING INTERESTS

None declared.

AUTHOR STATEMENT

DDS, PI, CH, HM-A and JVS designed the study. DDS and DT led the data collection. HM-A took part in the piloting. DDS led the data analysis and wrote the manuscript with input from all coauthors. All authors agreed on the final version of the manuscript.

DATA SHARING

No data are available. This is a qualitative study of a small, specific population in two unique geographic regions of rural Cambodia. Making the full data set publicly available could potentially breach the privacy that was promised to participants when they agreed to take part and the ethics approval granted from the National Ethics Committee for Human Research in Cambodia and the Swedish Ethical Review Authority. Therefore, the authors will not make the full transcripts available to a wider audience.

REFERENCES

1 World Health Organization. Maintaining essential health services: operational guidance for the COVID-19 context. Geneva, Switzerland: 2020.

2 Buonsenso D, Cinicola B, Kallon MN, et al. Child Healthcare and Immunizations in Sub-Saharan Africa During the COVID-19 Pandemic. *Front Pediatr* 2020;**8**.

3 Kruk ME, Myers M, Varpilah ST, et al. What is a resilient health system? Lessons from Ebola. *Lancet* 2015;**385**:1910–2.

4 Haldane V, Ong S-E, Chuah FL-H, et al. Health systems resilience: meaningful construct or catchphrase? *Lancet* 2017;**389**:1513.

For peer review only - http://bmjopen.bmj.com/site/about/guidelines.xhtml

5 Barasa EW, Cloete K, Gilson L. From bouncing back, to nurturing emergence: reframing the concept of resilience in health systems strengthening. *Health Policy Plan*. 2017;**32**:4.

6 IPCC. Global warming of 1.5°C. An IPCC Special Report on the impacts of global warming of 1.5°C above pre-industrial levels and related global greenhouse gas emission pathways, in the context of strengthening the global response to the threat of climate change, sustainable development, and efforts to eradicate poverty. Geneva, Switzerland: IPCC. 2018.

7 Watts N, Adger WN, Agnolucci P, et al. Health and climate change: policy responses to protect public health. *Lancet* 2015;**386**:1861–914.

8 Watts N, Amann M, Ayeb-Karlsson S, et al. The Lancet Countdown on health and climate change: from 25 years of inaction to a global transformation for public health. *Lancet* 2018;**391**:581–630.

9 Whitmee S, Haines A, Beyrer C, et al. Safeguarding human health in the Anthropocene epoch: report of The Rockefeller Foundation–Lancet Commission on planetary health. *Lancet* 2015;**386**:1973–2028.

10 Curtis S, Fair A, Wistow J, et al. Impact of extreme weather events and climate change for health and social care systems. *Environ Health*. 2017;**16**(Suppl 1):128.

11 Salas RN, Jha AK. Climate change threatens the achievement of effective universal healthcare. *BMJ* 2019;**366**:l5302.

12 Hanefeld J, Mayhew S, Legido-Quigley H, *et al.* Towards an understanding of resilience: responding to health systems shocks. *Health Policy Plan* 2018;**33**:355–67.

13 Barasa E, Mbau R, Gilson L. What Is Resilience and How Can It Be Nurtured? A Systematic Review of Empirical Literature on Organizational Resilience. *Int J Health Policy Manag*. 2018;**7**:491–503.

14 Turenne CP, Gautier L, Degroote S, *et al.* Conceptual analysis of health systems resilience: A scoping review. *Soc Sci Med* 2019;**1**:168–80.

15 Blanchet K, Nam SL, Ramalingam B, *et al.* Governance and capacity to manage resilience of health systems: Towards a new conceptual framework. *Int J Health Policy Manag*. 2017;**6**:5.

16 Thomas S, Keegan C, Barry S, *et al.* A framework for assessing health system resilience in an economic crisis: Ireland as a test case. *BMC Health Serv Res* 2013;**13**:450.

17 OECD. Guidelines for resilience systems analysis. Paris, France: Organisation for Economic Co-operation and Development. 2014.

18 Naimoli JF, Saxena S. Realizing their potential to become learning organizations to foster health system resilience: opportunities and challenges for health ministries in low- and middle-income countries. *Health Policy Plan* 2018;**33**:1083–95. 0

19 Gilson L, Barasa E, Nxumalo N, *et al.* Everyday resilience in district health systems: emerging insights from the front lines in Kenya and South Africa. *BMJ Glob Health* 2017;**2**:e000224.

20 Ministry of Health. National Strategic Plan on Disaster Risk Management for Health, 2015-2019. Phnom Penh, Cambodia: Ministry of Health. 2015.

For peer review only

21 National Committee for Disaster Management, Ministry of Planning. Strategic National Action Plan for Disaster Risk Reduction: 2008-2013. Phnom Penh, Cambodia: 2008.

22 World Food Programme. 2012 Cambodia post-flood relief and recovery survey. Phnom Penh, Cambodia: WFP 2012.

23 Ministry of Health Cambodia. Climate Change Strategic Plan for Public Health: Towards increased climate resilience for better health and well-being of all Cambodians. Phnom Penh, Cambodia: 2013.

24 Annear PL, Grundy J, Ir P, *et al.* The Kingdom of Cambodia Health System Review. Asia Pacific Observatory on Public Health Systems and Policies 2015.

25 Sochas L, Channon AA, Nam S. Counting indirect crisis-related deaths in the context of a low-resilience health system: the case of maternal and neonatal health during the Ebola epidemic in Sierra Leone. *Health Policy Plan* 2017;**32**:iii32–9.

26 Kruk ME, Kujawski S, Moyer CA, *et al.* Next generation maternal health: external shocks and health-system innovations. *Lancet* 2016;**388**:2296–306.

27 Royal Government of Cambodia. National Action Plan for Disaster Risk Reduction 2014-2018. Phnom Penh, Cambodia: Royal Government of Cambodia 2015.

28 Ministry of Health. Guidelines on Minimum Package of Activities for health center development 2008-2015. Phnom Penh, Cambodia: Ministry of Health 2007.

29 Ministry of Health. National guidelines on Complementary Package of Activities for referral hospital development from 2006 to 2010. Phnom Penh, Cambodia: Ministry of Health 2006.

30 Saulnier DD, Hanson C, Ir P, *et al.* The effect of seasonal flooding on health: Analysis of six years of health data in a flood-prone setting. *Int J Environ Res Public Health* 2018;**15**:pii:E656.

31 Malterud K, Siersma VD, Guassora AD. Sample Size in Qualitative Interview Studies: Guided by Information Power. *Qual Health Res* 2016;**26**:1753–60.

32 Braun V, Clarke V. Using thematic analysis in psychology. *Qual Res Psychol*. 2006;**3**:77–101.

33 Bene C, Wood RG, Newsham A, *et al.* IDS Working Paper 405. Resilience: New Utopia or New Tyranny? Reflection about the potentials and limits of the concept of resilience in relation to vulnerability reduction programmes. Brighton, UK: Institute of Development Studies, 2012.

34 Witter S, Wurie H, Chandiwana P, *et al.* How do health workers experience and cope with shocks? Learning from four fragile and conflict-affected health systems in Uganda, Sierra Leone, Zimbabwe and Cambodia. *Health Policy Plan* 2017;**32**:iii3–13.

35 Alonso-Garbayo A, Raven J, Theobald S, *et al.* Decision space for health workforce management in decentralized settings: a case study in Uganda. Health Policy Plan. 2017;**32**:iii59–66.

36 Kelsall T, Heng S. Inclusive healthcare and the political settlement in Cambodia. *New Polit Econ* 2016;**21**:238–55.

37 Liverani M, Chheng K, Parkhurst J. The making of evidence-informed health policy in Cambodia: knowledge, institutions and processes. *BMJ Glob Health* 2018;**3**:e000652.

For peer review only

For peer review only - http://bmjopen.bmj.com/site/about/guidelines.xhtml

38 Saulnier DD, Hean H, Thol D, *et al.* Staying afloat: community perspectives on health system resilience in the management of pregnancy and childbirth care during floods in Cambodia. *BMJ Glob Health* 2020;**5**.

39 Kittelsen SK, Keating VC. Rational trust in resilient health systems. *Health Policy Plan* 2019;**34**:553–7.

40 Kruk ME, Ling EJ, Bitton A, *et al.* Building resilient health systems: a proposal for a resilience index. *BMJ* 2017;**357**:j2323.

41 RESYST. Using Intersectionality to better understand health system resilience. London, UK: London School of Hygiene and Tropical Medicine 2017.

For peer review only

For peer review only - http://bmjopen.bmj.com/site/about/guidelines.xhtml

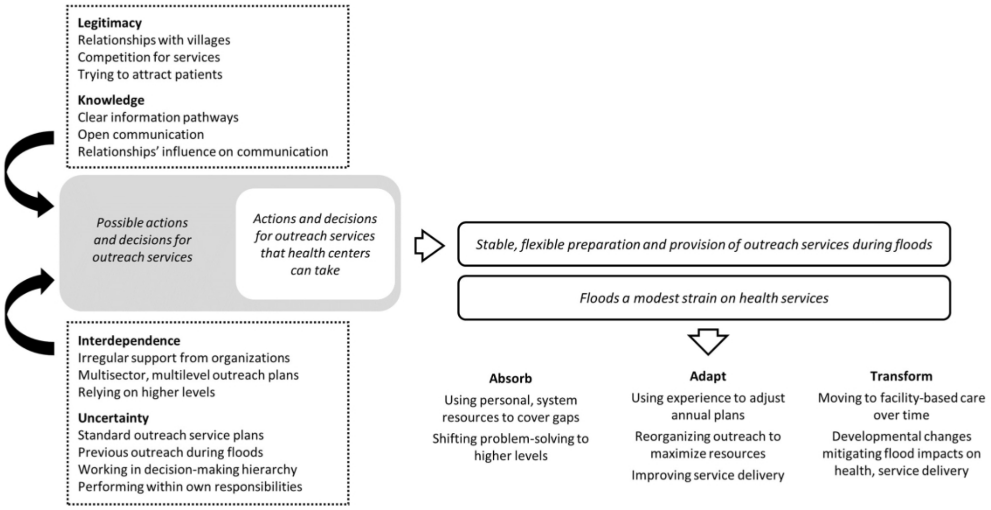

Figure 1. A visual representation of the four dimensions of resilience and the system's capacity to absorb, adapt, and transform as they relate to the main theme, using health center outreach services for antenatal and childbirth care during a flood as an example

196x104mm (300 x 300 DPI)

Supplement 1. Topic guide in English for semi-structured interviews with health facility providers or health department staff.

Flood experiences

Give example of recent flood in the area and confirm event (when, where, length of flood). Can you tell me about the last flood that happened near your [facility/catchment area]?

Explore: Effect on villages and health

Can you describe your experiences of working during a flood?

Explore: Changes/differences in care compared to no floods

Provision and maintenance of services

When there is a flood, what happens to prenatal care services at your [facility/catchment area]? What happens to delivery care services?

Explore: Demand and access to care, staff changes, supplies, medicines, user fees, budget, management from upper levels

Can you describe a time when a woman was not able to get prenatal care at a [facility] during a flood? What happened? What about a time when a woman was not able to get delivery care at a [facility]? What happened then?

Anticipating and coping with uncertainty

How do you prepare prenatal services for the rainy season? How do you prepare delivery services?

Can you share specific examples of things that you do at the [facility/health department] to make sure that pregnant women are able to continue getting care during floods?

External factors influencing the health system

What are some reasons that your [facility/health department] might not be able to provide prenatal care during floods? What about for delivery care?

Explore: Support and work with other departments/sectors/NGOs/committees, transport and access to facilities, supply chain, available funds, staff personal lives and priorities, changes in health, emergencies

What do you think influences pregnant women to come to your facility for prenatal care during floods? What about for delivery care?

Interaction with the community

How does the [facility/health department] work with pregnant women in the village during floods?

Explore: Reasons for visiting other providers or home delivery, outreach in villages, input and accountability with community

In your opinion, how do you think the pregnant women feel about the prenatal care that they can get at [facilities] during floods? What about delivery care?

Explore: Trust and quality, social media, feeling of ownership

Gathering and using knowledge

What kind of decisions do you have to make about services for prenatal care during floods? For delivery care?

Explore: Referring patients to hospital, emergency obstetric cases, sources of information, communication with other departments/facilities/committees, flexibility and ability to make decisions

From your experience working during floods, what have you learned about providing prenatal care during floods? What have you learned about providing delivery care?

Can you give me an example of something you would like to know when there is a flood that would help make prenatal care better during floods? And for delivery care?

For peer review only

COREQ (COnsolidated criteria for REporting Qualitative research) Checklist

A checklist of items that should be included in reports of qualitative research. You must report the page number in your manuscript where you consider each of the items listed in this checklist. If you have not included this information, either revise your manuscript accordingly before submitting or note N/A.

Topic	Item No.	Guide Questions/Description	Reported on Page No.
**Domain 1: Research team and reflexivity**			
Personal characteristics			
Interviewer/facilitator	1	Which author/s conducted the interview or focus group?	6
Credentials	2	What were the researcher's credentials? E.g. PhD, MD	6
Occupation	3	What was their occupation at the time of the study?	6
Gender	4	Was the researcher male or female?	6
Experience and training	5	What experience or training did the researcher have?	6
Relationship with participants			
Relationship established	6	Was a relationship established prior to study commencement?	6
Participant knowledge of the interviewer	7	What did the participants know about the researcher? e.g. personal goals, reasons for doing the research	6
Interviewer characteristics	8	What characteristics were reported about the inter viewer/facilitator? e.g. Bias, assumptions, reasons and interests in the research topic	6
**Domain 2: Study design**			
Theoretical framework			
Methodological orientation and Theory	9	What methodological orientation was stated to underpin the study? e.g. grounded theory, discourse analysis, ethnography, phenomenology, content analysis	6
Participant selection			
Sampling	10	How were participants selected? e.g. purposive, convenience, consecutive, snowball	6
Method of approach	11	How were participants approached? e.g. face-to-face, telephone, mail, email	6
Sample size	12	How many participants were in the study?	6
Non-participation	13	How many people refused to participate or dropped out? Reasons?	7
Setting			
Setting of data collection	14	Where was the data collected? e.g. home, clinic, workplace	6
Presence of non-participants	15	Was anyone else present besides the participants and researchers?	6
Description of sample	16	What are the important characteristics of the sample? e.g. demographic data, date	6
Data collection			
Interview guide	17	Were questions, prompts, guides provided by the authors? Was it pilot tested?	6
Repeat interviews	18	Were repeat inter views carried out? If yes, how many?	6
Audio/visual recording	19	Did the research use audio or visual recording to collect the data?	6
Field notes	20	Were field notes made during and/or after the inter view or focus group?	6
Duration	21	What was the duration of the inter views or focus group?	6
Data saturation	22	Was data saturation discussed?	6
Transcripts returned	23	Were transcripts returned to participants for comment and/or	6

For peer review only

For peer review only - http://bmjopen.bmj.com/site/about/guidelines.xhtml

Topic	Item No.	Guide Questions/Description	Reported on Page No.
		correction?	
**Domain 3: analysis and findings**			
Data analysis			
Number of data coders	24	How many data coders coded the data?	7
Description of the coding tree	25	Did authors provide a description of the coding tree?	7
Derivation of themes	26	Were themes identified in advance or derived from the data?	7
Software	27	What software, if applicable, was used to manage the data?	7
Participant checking	28	Did participants provide feedback on the findings?	7
Reporting			
Quotations presented	29	Were participant quotations presented to illustrate the themes/findings? Was each quotation identified? e.g. participant number	7-12
Data and findings consistent	30	Was there consistency between the data presented and the findings?	7-12
Clarity of major themes	31	Were major themes clearly presented in the findings?	7-12
Clarity of minor themes	32	Is there a description of diverse cases or discussion of minor themes?	7-12

Developed from: Tong A, Sainsbury P, Craig J. Consolidated criteria for reporting qualitative research (COREQ): a 32-item checklist for interviews and focus groups. *International Journal for Quality in Health Care*. 2007. Volume 19, Number 6: pp. 349 – 357

Once you have completed this checklist, please save a copy and upload it as part of your submission. DO NOT include this checklist as part of the main manuscript document. It must be uploaded as a separate file.

For peer review only

BMJ Open

'We have a plan for that': A qualitative study of health systems resilience through the perspective of health workers managing antenatal and childbirth services during floods in Cambodia

Journal:	*BMJ Open*
Manuscript ID	bmjopen-2021-054145.R1
Article Type:	Original research
Date Submitted by the Author:	09-Nov-2021
Complete List of Authors:	Saulnier, Dell; Karolinska Institutet, Department of Global Public Health<br>Thol, Dawin; National Institute for Mental Health<br>Por, Ir; National Institute of Public Health<br>Hanson, Claudia; Karolinska Institutet, Department of Global Public Health; London School of Hygiene & Tropical Medicine<br>Von Schreeb, Johan ; Karolinska Institutet, Department of Global Public Health<br>Alvesson, Helle; Karolinska Institutet, Department of Global Public Health
**Primary Subject Heading**:	Global health
Secondary Subject Heading:	Qualitative research, Health services research
Keywords:	HEALTH SERVICES ADMINISTRATION & MANAGEMENT, Change management < HEALTH SERVICES ADMINISTRATION & MANAGEMENT, QUALITATIVE RESEARCH, PUBLIC HEALTH

SCHOLARONE™
Manuscripts

I, the Submitting Author has the right to grant and does grant on behalf of all authors of the Work (as defined in the below author licence), an exclusive licence and/or a non-exclusive licence for contributions from authors who are: i) UK Crown employees; ii) where BMJ has agreed a CC-BY licence shall apply, and/or iii) in accordance with the terms applicable for US Federal Government officers or employees acting as part of their official duties; on a worldwide, perpetual, irrevocable, royalty-free basis to BMJ Publishing Group Ltd ("BMJ") its licensees and where the relevant Journal is co-owned by BMJ to the co-owners of the Journal, to publish the Work in this journal and any other BMJ products and to exploit all rights, as set out in our licence.

The Submitting Author accepts and understands that any supply made under these terms is made by BMJ to the Submitting Author unless you are acting as an employee on behalf of your employer or a postgraduate student of an affiliated institution which is paying any applicable article publishing charge ("APC") for Open Access articles. Where the Submitting Author wishes to make the Work available on an Open Access basis (and intends to pay the relevant APC), the terms of reuse of such Open Access shall be governed by a Creative Commons licence – details of these licences and which Creative Commons licence will apply to this Work are set out in our licence referred to above.

Other than as permitted in any relevant BMJ Author's Self Archiving Policies, I confirm this Work has not been accepted for publication elsewhere, is not being considered for publication elsewhere and does not duplicate material already published. I confirm all authors consent to publication of this Work and authorise the granting of this licence.

For peer review only

Title

'We have a plan for that': A qualitative study of health systems resilience through the perspective of health workers managing antenatal and childbirth services during floods in Cambodia

Authors

1. Dell D Saulnier, PhD – Department of Global Public Health, Karolinska Institutet, Stockholm, Sweden.

   Corresponding author address: Karolinska Institutet, Widerströmska huset, Plan 3, Tomtebodavägen 18a, 171 77 Stockholm, Sweden. *Phone*: +46 733 232 076. *Email:* dell.saulnier@ki.se
2. Thol Dawin, MD, MPH - National Institute of Public Health, Phnom Penh, Cambodia
3. Ir Por, MD, PhD - National Institute of Public Health, Phnom Penh, Cambodia
4. Claudia Hanson, MD, PhD - Department of Global Public Health, Karolinska Institutet; Department of Disease Control, London School of Hygiene and Tropical Medicine
5. Johan von Schreeb, MD, PhD - Department of Global Public Health, Karolinska Institutet
6. Helle Mölsted Alvesson, PhD - Department of Global Public Health, Karolinska Institutet

Keywords

Health systems resilience; floods; shocks; Cambodia; health service provision

Word count: 4 057

For peer review only - http://bmjopen.bmj.com/site/about/guidelines.xhtml

ABSTRACT

Objective Health systems resilience can increase a system's ability to deal with shocks like floods. Studying health systems that currently exhibit the capacity for resilience when shocked could enhance our understanding about what generates and influences resilience. This study aimed to generate empirical knowledge on health systems resilience by exploring how public antenatal and childbirth health services in Cambodia have absorbed, adapted, or transformed in response to seasonal and occasional floods.

Design A qualitative study using semi-structured interviews and thematic analysis and informed by the Dimensions of Resilience Governance framework.

Setting Public sector healthcare facilities and health departments in two districts exposed to flooding

Participants Twenty-three public sector health professionals with experience providing or managing these services during recent flooding.

Results The theme 'Collaboration across the system creates adaptability in the response' reflects how collaboration and social relationships among providers, staff, and the community have delineated boundaries for actions and decisions for antenatal and childbirth care during floods. Floods were perceived as having a modest impact on health services. Knowing the boundaries on decision-making and having preparation and response plans let staff prepare and respond in a flexible yet stable way. The theme was derived from ideas of: i) seasonal floods as a minor strain on the system compared to persistent, system-wide organizational stresses the system already experiecnes, ii) the ability of the health services to adjust and adapt flood plans, iii) a shared purpose and working process during floods, iv) engagement at the local level to fulfill a professional duty to the community, and v) creating relationships between health system levels and the community to enable flood response.

Conclusion The capacity to absorb and adapt to floods was seen among the public sector services. Strategies that enhance stability and flexibility may foster the capacity for health systems resilience.

ARTICLE SUMMARY

Strengths and limitations of the study

For peer review only - http://bmjopen.bmj.com/site/about/guidelines.xhtml

- Participants in a variety of roles and from multiple levels of the health system strengthened the diversity of perspectives on health service functioning during floods.
- Extensive discussion among the diverse research team following all interviews and a comprehensive audit trail increased the dependability and credibility of the study.
- The data collectors were familiar with rural Cambodia, its health system, and health services at multiple levels, which helped the quality of the interviews.
- Participants at higher levels of authority may have provided more socially desirable information or refrained from sharing the full extent of their experiences or thoughts.
- The participants' rationale for their actions when preparing and responding to floods was not always articulated, which may affect the reliability of the results.

For peer review only - http://bmjopen.bmj.com/site/about/guidelines.xhtml

INTRODUCTION

Since early 2020, health systems worldwide have been severely challenged by the COVID-19 pandemic. Systems have faced a sudden demand to surveille, diagnose, and care for people with COVID-19 and the continued demand for essential services like vaccination and maternal healthcare;[1] in some cases, the pandemic has already disrupted these services.[2] Attention has been increasingly placed on health systems resilience as a way to reduce health system vulnerability and increase their ability to deal with shocks like the 2014-2016 West Africa Ebola outbreak, the COVID-19 pandemic, or extreme weather events.[3–5]

Extreme weather events are projected to become more frequent and severe as a consequence of climate change, including an increase in seasonal floods and occasional floods.[6] By the end of the century, an additional 2.3 billion people are expected to be at risk of flooding.[7] The health impacts of seasonal and occasional floods are diverse, from direct mortality and morbidity to indirect poor health from their impact on societal functioning.[8,9] Health systems, crucial to managing the health of the population, are also directly and indirectly impacted by floods.[9–11]

Resilient health systems have the capacity to continue delivering health services when shocked or stressed, responding and adapting to the new health needs created by the shock or stress and the routine health needs of the population.[3,12,13] Systems are exposed to shocks, sudden or extreme external phenomena that impact healthcare demand and health system resources, and chronic, internal stresses that continually challenge the system's functioning, like persistent underfunding.[14] Health systems that are exposed to severe shocks will need to undergo a greater degree of change in order to be resilience than systems exposed to less severe shocks and stresses.[15]

To date, the literature has focused on how to define and understand the concept of health systems resilience; less has been published on how resilience can be generated.[5,14,16] Exploring what makes health systems resilient in real-world situations is necessary to understand what capacities could foster resilience to shocks like floods.

For peer review only - http://bmjopen.bmj.com/site/about/guidelines.xhtml

Cambodia is regularly exposed to repeated seasonal and occasional floods, as well as flood disasters. Improving health service delivery readiness to operate during floods has been an ongoing priority for the government of Cambodia since 2008,[17,18] and the health services have remained functional during previous flood disasters.[19] Concurrent with reforms that have focused on strengthening health service delivery and management, several strategic plans have been developed to enhance the health system's ability to respond to floods.[17,20,21] This study therefore aims to understand how health system resilience is generated, by exploring the capacity of the Cambodian health system to absorb, adapt, and transform during floods. We sought to understand how healthcare workers in public sector health facilities and health departments are able to continue providing health services during seasonal and occasional floods, using antenatal and childbirth services as indicators of routine and new health needs, respectively.[22,23]

Conceptual framework

The study was designed using the Dimensions of Resilience Governance framework.[24] The framework argues that a health system's ability to manage shocks and stress is dependent on the ability of the system and the people within it to manage four other capacities: i) the ability to access, integrate, and process *knowledge* from inside and outside the health system, ii) the ability to adapt in response by taking actions and decisions to anticipate and cope with *uncertainty*; iii) the capacity to manage interactions at multiple levels of the health system and with other systems (*interdependence*), and iv) the capacity to engage with users and communities to create a socially and contextually accepted system (*legitimacy*).[24] Health systems can then absorb shocks or stress using existing strategies and resources, adapt to them by temporarily adjusting how resources are used, or transform the structure of the system in the long-term to avoid them altogether.[5,25,26]

METHODS

Study setting

For peer review only - http://bmjopen.bmj.com/site/about/guidelines.xhtml

Cambodia is a predominantly rural, lower middle-income country. Rapid economic and developmental changes increased the life expectancy from 58.4 to 68.6 years between 2000 to 2015, and maternal health and service indicators have been improving over the last 20 years (Table 1).[21,27] The country experiences annual flooding along the main rivers and Tonle Sap lake and occasional floods from monsoon rains in other regions. The National Committee for Disaster Management leads a national-to-district level program for disaster management and flood response.[28] Operational preparedness and response plans exist for multiple levels of the health system and include items such as roles and responsibilities during disasters (Box 1).

Box 1. Existing preparedness and response measures for antenatal and childbirth services during floods (Ministry of Health 2015)

The 2015 strategic plan for health during disasters prioritized building capacity for disaster risk reduction, by strengthening governance and coordination between sectors and actors during emergencies, improving information and knowledge management, and bolstering health service delivery and resources during disasters. Preparedness and response plans for health services during floods have been created for each province, based on post-flood reviews and local knowledge of high-risk flood areas and the facilities within them. The plans include strategies such as stockpiling resources, surge capacity protocols, and contingency budgets. Maternal health services were identified as an essential service at risk during floods, and specific strategies for antenatal and birth services include improving midwifery skills in general prior to flooding, providing medical kits with the relevant equipment to mobile outreach teams, and ensuring pregnant women can be identified and evacuated during floods, if needed.

The study used antenatal and childbirth services to explore service provision. These services are indicative of how health services for new and routine health needs function during floods, even though neither pregnancy nor childbirth or emergency pregnancy complications are causally linked to floods. Pregnancy is representative of routine health needs that do not change because of floods but still require preventive and promotive care that can be planned in advance, while deliveries and complications are representative of new health needs that can occur, sometimes unexpectedly, during floods and require skilled management and emergency care[33,34].

In the Cambodian health system, districts link health centers and district hospitals to the provincial level through health departments, which oversee service delivery and provides operational support. During floods, public health facilities conduct additional health promotion, education, and clinical outreach, and

health departments prepare resources and oversee the flood response. Routine antenatal and uncomplicated childbirth care is provided at public health centers and private clinics. Additional antenatal services and emergency obstetric care is provided at private clinics and public hospitals.[29]

Table 1. Maternal health and health service indicators in Cambodia [30–32]

	**2000**	**2017**
Maternal mortality ratio (deaths per 100 000 live births)	488	160
	**2000**	**2014**
Percent of women receiving antenatal care at least once	43.2	95.2
Percent of antenatal care received at a public facility	---	94.0
Percent of childbirth care at a healthcare facility	9.9	83.2
Percent of childbirth care at a public facility	---	68.9
Percent of childbirth care at home	89.0	16.6
Percent of childbirths attended by a doctor, nurse, or midwife	31.8	89.0
Percent of childbirths by caesarean section	0.8	6.3

Study design, participants, and recruitment

This qualitative study used semi-structured interviews to explore public sector healthcare providers and health department staff experiences in delivering and managing antenatal health services during floods. Two districts were selected: one that typically floods every year (seasonal flooding) and one that experiences floods less often (occasional flooding) to cover the main frequencies of flooding in Cambodia.[33] The study included five public health centers serving a population of 10 000 each, two district referral hospitals serving populations of 200 000 people, two provincial-level and two district-level health departments, two communes, and the Ministry of Health . The history of flooding in the provinces and districts was first discussed with the health departments to confirm recent flooding and obtain access to the health centers. The recently flooded health centers were then visited and enrolled after confirming that their catchment areas had flooded in the previous five months. Participants who had been working in their current role during the most recent flood were purposively selected for variety type of job, length of

For peer review only - http://bmjopen.bmj.com/site/about/guidelines.xhtml

experience in their job, age, and gender.(Table 2). All health center and hospital chiefs were invited to participate and identified eligible midwives at their facilities. Health department and commune chiefs identified eligible participants among their staff that were invited to participate.

Data collection

Two female Cambodian data collectors (a midwife studying public health and a qualitative researcher and retired medical school lecturer) conducted and audio recorded 23 semi-structured interviews in Khmer. Interviews were conducted in November and December 2018 at the end of the rainy season to ensure that detailed experiences with recent flooding could be captured in the discussions. Participation was voluntary and unpaid, and all participants gave written or recorded verbal consent prior to the interviews. The interview guides (Supplement 1) were used to investigate participants' real-life experiences working with service provision and management during floods. Interview guide questions were developed from the four capacities in the Dimensions of Resilience Governance framework [24] which were adapted into concrete questions about the participants' experiences. Participants were interviewed individually in private areas such as empty meeting rooms. The second data collector had taught five of the participants in medical school since the 1980s; she acknowledged the connections and kept the interview focused on the interview guides. The quality of the interviews was assessed continually among the team through discussion and field notes. The 23 interviews were judged as sufficient to have captured the relevant information based on the information power concept, which states that samples that hold a high degree of relevant information require a smaller number of participants.[34] Interviews were transcribed verbatim into Khmer and translated into English by one translator. Uncertainties and inconsistencies between the transcripts and audio files during translation were discussed with the data collectors and the first author.

Table 2. Characteristics of the study sites and participants

Source	*District*	*Summary of most recent flood*	*Professional area*	*Years in current role*	*Interview length (minutes)*

For peer review only - http://bmjopen.bmj.com/site/about/guidelines.xhtml

Ministry of Health	--	--	Disaster management	13	58
Provincial health department	Seasonal	June to October; one-third of districts flooded	Maternal health	15	57
			Disaster management	3	61
Provincial health department	Occasional	July; half of districts flooded	Maternal health	1	75
			Disaster management	3	64
District health department	Seasonal	June to October; flooding around all health centers	Disaster management	22	81
District health department	Occasional	Unsure of dates; flooding around 3 health centers	Disaster management	22	83
Commune	Seasonal	July to October; in villages	Deputy commune chief	4	61
Commune	Occasional	August to October; in villages	Committee for Women's Affairs	15	77
Referral hospital	Seasonal	June to October; flooded villages, not near hospital	Hospital chief	12	45
			Midwife	6	47
Referral hospital	Occasional	July to August; flooded villages, not near hospital	Health center chief	19	36
			Midwife	4	45
Health center 1	Seasonal	June to October; all villages in catchment flooded	Health center chief	6 months	58
			Midwife	6	50
Health center 2	Seasonal	August to October	Health center chief	1	64
			Midwife	28	61
Health center 3	Occasional	September to November; half of villages in catchment area flooded	Health center chief	3	61
			Midwife	3	74
Health center 4	Occasional	August to October; 2 of 7 villages in catchment area flooded	Health center chief	10	53
			Midwife	4	77
Health center 5	Occasional	October to November; 2 of 7 villages in catchment area flooded	Health center chief	18	57
			Midwife	8	97

Data analysis

A data-driven thematic analysis [35], where codes are chosen based on a detailed analysis of the data rather than based on theory, was conducted in Nvivo 12 Pro. It was chosen to ensure that the theoretical concepts of the framework did not eclipse other relevant information in the data about the health system's functioning and to capture ideas that spanned multiple dimensions in the framework. Codes were developed by DS that reflected descriptions or ideas about influences on health service delivery during

For peer review only - http://bmjopen.bmj.com/site/about/guidelines.xhtml

floods. After coding was complete, the authors explored how the capacities of knowledge, uncertainty, interdependence, and legitimacy from the framework could be influencing the relationship between codes. Categories were then created from the patterns of meaning from the data-driven coding plus the theoretical understanding gained during the previous step and a single theme was identified that captured the meaning and association between the categories. Lastly, the findings were mapped back onto the framework to illustrate how they contributed to the framework's capacities (Figure 1)

Patient and public involvement

No patients involved.

Ethical considerations

The National Ethics Committee for Human Research in Cambodia (ref: 204 and 276) and the Swedish Ethical Review Authority (Dnr: 2019-02458) approved the study. One participant requested that part of their interview be excluded from transcription and analysis.

RESULTS

We identified one theme, 'Collaboration across the system creates adaptability in the response', derived from five categories (Table 3). Collaboration and social relationships appeared to create clear boundaries to decision-making around antenatal and birth care during floods. Both seasonal and occasional floods were discussed as strains rather than acute shocks or chronic stresses. With a firm understanding of the decision-making boundaries, providers and staff were able to prepare and respond to these floods in a flexible but stable manner, resulting in absorptive and adaptive capacity. Overarching health system and contextual changes appear to be fundamentally changing public sector service use and function during floods, potentially transforming the system and the impact of floods themselves.

Table 3. The theme, categories, and subcategories describing the influences on public sector antenatal and childbirth health services during floods

Theme	Categories	Sub-categories
Collaboration	Floods just another strain on service delivery	

For peer review only - http://bmjopen.bmj.com/site/about/guidelines.xhtml

across the system creates adaptability in the response	Facilities and health departments able to calibrate and maneuver to make flood routines work for them	
	Working in the same direction during floods	
	Engaging in local governance to fulfill a duty to the community during floods	Health centers becoming obsolete during floods<br>Believing in the value of the system during floods
	Creating relationships to successfully respond to floods	Health centers as the lynchpin for the health response to floods<br>Social relationships can challenge information sharing

We illustrate the relationship between the theme and the Dimensions of Resilience Governance framework in Figure 1, followed by a description of the categories informing the theme. The four dimensions feed into the process outlined in the theme, showing how boundaries on decision-making are contributing to preparation and response to modest seasonal and occasional floods, helping to create the capacity to absorb, adapt, and transform.

Figure 1. A visual representation of the four dimensions of resilience and the system's capacity to absorb, adapt, and transform as they relate to the main theme, using health center outreach services for antenatal and childbirth care during a flood as an example

'Floods just another strain on services'

Participants did not perceive seasonal or occasional floods as a threat to health service functioning. Instead, they were part of a larger constellation of constant pressures on service delivery. Providers and health department staff in both districts described pregnancy, childbirth, and other health problems during floods as routine and "nothing serious". They note that antenatal and childbirth services usually functioned as normal during both types of floods. Changes over time, like better roads, let participants feel that floods were no longer a major threat:

For peer review only

The floods are not so big that we have to focus on them so much. They're not big to the point of an emergency. (IDI 7, provincial health department)

Participants described numerous, persistent challenges to service delivery that were independent from flooding. They included i) slow, unreliable referral systems, ii) inadequate professional training, iii) heavy workloads and staff shortages, iv) unintegrated and undependable support from not-for-profit organizations, v) poor or inaccurate health and system performance information, and vi) a slow and unresponsive process for requesting funds, resources, and help from higher levels of the health system. Floods exacerbated these challenges but everyday strains on service provision subsumed the effect of floods:

There's no shortage of medicine [during floods]. The hospital had bought and stored it at the hospital so that there are no shortages happening. It's just that there's no leftover money to encourage the staff. (IDI 20, referral hospital midwife)

'Facilities and health departments able to calibrate and maneuver to make flood routines work for them'

Staff at facilities and departments reviewed the standard flood response plans each year. Together with flood warnings and their experiences from previous floods, participants in both districts discussed how they knew the routines for preparing. They began to actively prepare in the months and weeks preceding the expected floods:

All the plans are already written […] We have planned what to do, we have all events organized, and we just follow accordingly. We have written everything into the yearly plan, arranged it by month as well. And if there's a flood, we have listed down already what we need to do, what to buy, and so on. (IDI 2, health center chief)

According to participants, facilities and health departments in both districts calibrated their plans and routines to fit different scenarios, and flexibly manoeuvred during flood response. They adapted the standardized plans—formulated for disasters—to fit large- and smaller-scale floods in each district. The adaptions were based on previous experience and scenarios they might encounter. The plans used

For peer review only - http://bmjopen.bmj.com/site/about/guidelines.xhtml

strategies like: i) diverting clinical staff between facilities and health departments to cover shortages or supplement outreach teams, ii) using discretionary budgets to prepare or respond, iii) piggybacking on the non-health response to floods, and iv) choosing how to organize outreach for flood-related care:

> *We have a boat, it's even a motorboat. It costs more to transport the boat to the [flooded] area than it does to rent a local boat there. We ride a motorbike and then ride on the boat with the locals. It costs less.* (IDI 18, district health department)

'Working in the same direction during floods'

Facility chiefs and health department staff described a multilevel, multisectoral flood response in both districts. Bigger floods required a larger response and more organization at the provincial level with external sectors. Participants knew that they could not control problems outside the health sector that affected health or their work during floods and needed to trust other sectors and higher authorities to find solutions:

> *There're only the commune and the district that could solve the problem [of difficult travel during floods]. They [facility staff] can only complain to us but we can't do anything about it at all. For problems regarding the road, only the commune can make a plan and ask that the province supports it.* (IDI 8, referral hospital midwife)

Participants noted that they had to collaborate between levels of the health system to keep facility-based services and clinical outreach functional during floods. They relied on higher organizational levels of the health system to be responsible for problem-solving or to help with supplies, staff, and expertise; were free and able to contact higher levels for help, and described a strong sense of teamwork across levels when responding to floods:

> *When there's a big flood, the district will have to visit the flooded areas every day to see the people's situation, how they are living, is there any clean water for them to use, any diarrhea, are there any health center staff to help them with their health problems or not? Everyone is enthusiastic when there's a big flood, both the provincial level and district level.* (IDI 10, district health department)

For peer review only - http://bmjopen.bmj.com/site/about/guidelines.xhtml

While free to ask for help, participants described clear limits to their decision-making. They stayed within the boundaries of their designated role when preparing and responding to floods. Their superiors made and approved most decisions about responding to bigger floods, noted in particular by midwives and health department staff:

> *The decisions that I can make during a disaster without asking the upper level to help are none. During a disaster, you have to depend on the upper level. I have to report to them because we cannot work alone.* (IDI 9, provincial health department)

'Creating relationships to successfully respond to floods'

Health department staff and providers were able to share information and keep facility-based care and outreach services functioning during floods, facilitated by their relationships with each other and the community.

Health centers as the lynchpin for the health response to floods

Participants saw the existing relationships between health center staff and the community as crucial for providing health services during floods. Health center staff worked to keep outreach and facility-based services functioning smoothly during floods. For example, they would pay out-of-pocket for gasoline or reimburse village leaders for emergency phone calls. Health center staff felt that the community's knowledge of health risks and strategies to stay healthy during floods made it easier to provide services:

> *The health center doesn't have a boat, not even a tuk tuk [to transfer patients during floods]. The women need to have their own boats. The villagers are very smart. They know that they need to have a boat with them when they come for antenatal care.* (IDI 14, health center midwife)

Health centers and departments needed villages to supply information on their health and the severity of the floods in their area so that they could respond appropriately. According to participants, health centers needed to negotiate with commune and village leaders to allow health department staff to enter to villages during floods:

For peer review only - http://bmjopen.bmj.com/site/about/guidelines.xhtml

Before we go to any village, we inform the health center to inform [the local authorities]. If you don't inform them, they won't know what you're doing and there will be trouble. If it's before an election, you would have to be even more careful. (IDI 18, district health department)

Social relationships can challenge information sharing

Participants described flood and health information as accessible to staff across all levels of the health system, structured to flow up or down from the village to the national level. Contact networks were openly accessed, regardless of rank or role. Open communication and information sharing meant contact was often between individuals rather than roles:

Before they [the health center] transfers a case here, they will call us. They call the hospital, there's a telephone there. Sometimes they contact [the hospital chief] directly. Sometimes they call any staff that they know here. (IDI 20, referral hospital midwife)

Personal and social relationships could hamper access and information sharing between individuals. Lower-ranking staff in health departments and facilities, like midwives, only received information about how their facility would respond to floods on a 'need-to-know' basis. Participants explained that they were more willing to contact known, trusted individuals for information or help. Without trust, participants felt obligated to follow up on information themselves to ensure it was correct:

Sometimes, the information that is available is not clear. Then we have to go to the community for an assessment. Like in the past, we had information from the National Committee for Disaster Management that there were deaths [during the flood] but then when I went there, they [the health department] said there were none. So, it's hard.". (IDI 23, Ministry of Health)

'Engaging in local governance to fulfill a duty to the community during floods'

Staff at the health centers remained engaged in holding themselves accountable for the care they delivered. They described feeling a professional duty and responsibility to the community's health during floods, despite feeling progressively less relevant in a changing landscape.

Health centers becoming obsolete during floods

For peer review only - http://bmjopen.bmj.com/site/about/guidelines.xhtml

Participants described health centers as struggling to compete for services with private facilities and public hospitals regardless of floods. Health center providers noted more women now chose facilities with better services and resources and expressed difficulty understanding why they were losing relevance:

> *It's not because there's a flood or not [that they go to the hospital to deliver]. We care for them since the first month to the eight or ninth month, then they disappear. When it's an appointment date, they miss it. Then after four or fiver days, they carry the baby here. I ask where was the baby born? It was born at the public hospital. I ask why not at [my health center]? They wanted to give birth there. That's their choice.* (IDI 1, health center midwife)

Health center participants felt obligated to focus on facility-based services rather than outreach for flood-related care and felt tied to their facilities during floods. They had to prioritize staff, funds, and equipment for the health center, and felt less able to provide comprehensive antenatal outreach during floods:

> *We have only one electric generator that is used at the health center. So we cannot take that into the village [during outreach]. If we do, when people come to the health center for treatment, then we can't provide full services to them.* (IDI 4, health center midwife)

As a result, clinical outreach during floods began when community members could no longer reach the health centers and focused on preventing communicable diseases.

Believing in the value of the system during floods

During floods, health centers described attempts to increase demand at their facilities, using schemes like waiving services fees and promoting facility-based care. They actively attempted to improve service quality at their facilities, for instance by holding providers accountable for perceived poor treatment of patients.

Providers and health department staff felt pride in their work and a professional duty to serve the community, despite the persistent challenges described earlier. When faced with floods, they discussed sharing a common goal of helping the system work as it should, regardless of potential challenges. This echoed earlier expressions about relying on higher levels of the health system and the community to take responsibility:

For peer review only

Cases of [women] unable to deliver, we never have them. We don't have any issue with it because we have the network to help her on time. If there are any problems, the village and commune chiefs have my phone number and the district head's phone number, if the patient needs an ambulance to take her from home. (IDI 19, hospital chief)

DISCUSSION

This qualitative study highlights the importance of understanding the practices that enable health service provision during flooding. The results show an adjustable approach to preparing and responding to floods. The approach is grounded in collaboration and relationships across the system that set boundaries around actions and decisions, giving the system the capacity to adapt and absorb floods. This approach was similar between the seasonal and occasionally flooded districts, despite the difference in flood frequency. Health system strengthening initiatives and developmental changes in Cambodia, like reforms to promote and improve facility-based maternal health services and better roads, have transformed the health system context over time. The boundaries around actions and decisions, coupled with the transformative changes, had led to a system that is capable of maintaining pregnancy and childbirth services during floods, and one that views floods as strains rather than shocks.

The absorptive and adaptive capacities were characterized by the health facilities' ability to prepare and respond to seasonal and occasional floods in a stable but flexible way, with support from the health departments. Transformative capacity was not readily reported by the participants. Some changes in the structure or functioning of the system, such as the general shift to facility-based antenatal and childbirth care, were not explicitly linked to flooding. If the three capacities are seen as 'different perspectives of the same reality',[15] stability and flexibility are both necessary:[13,36] stability to cushion against shocks and let coordinated actions emerge, and flexibility to change and adapt. In our results, previous experience and planning created the stability to absorb and adapt, and the flexibility to continue adapting to the current flood if needed.

A driver of the stability and flexibility appeared to be the relationships between individuals and groups that delimited actions and decision-making for services during floods. Health systems are populated by

For peer review only - http://bmjopen.bmj.com/site/about/guidelines.xhtml

health workers and community members with the power to make choices, and the social networks, relationships, and collaborations among them have been identified as influencers of resilience.[13,15,26,36,37] In this study, relationships influenced how knowledge was collected and shared during floods. Being able to rely on higher levels and other providers or staff was fundamental to the interdependent preparation and response described by the participants. Understanding the boundaries of the decision-making space appears as a key component to taking action, as seen in a study of decision-making in Uganda.[38] In the context of the Cambodian health system, where decision-making is already hierarchical,[39,40] top-down leadership may have helped counteract uncertainty when responding to floods; during floods, participants focused on actions and decisions within their own responsibilities, shifting decisions beyond their remit upwards in the hierarchy. However, further work would be needed to understand and compare the effects of hierarchy on uncertainty and decision-making in nonhierarchical contexts.

The descriptions by health center providers of their close, positive relationships with local community members contrast with their descriptions of a steady decrease in visits to health centers. An earlier study on community management of pregnancy and childbirth during floods identified low degree of trust and sense of ownership in health centers among community members.[41] In this study, participants described trying to increase trust and demand for services year-round, regardless of floods. This could be linked to their perception of floods as a strain to the system rather than a shock. The main challenge identified during floods was not coping with or responding to health needs, but how the usual conditions of the public health system constrained the quality or quantity of care the public health system could give. Without a strong basis of trust and quality when floods were not present, there may have been fewer incentives for community members to visit public health centers during floods, when barriers to care were higher.[41] Still, the participants appeared to have a strongly anchored belief in their roles and the care they provide, and were able to build relationships with community members, which may be seeds for constructing a more legitimate system.[12,37,42,43]

For peer review only

Further differentiating the concepts of shocks and stresses could be useful to understand how characteristics of resilience might vary depending on what challenges a system faces.[13,44] Both seasonal and occasional floods were described as a strain on the health system—acute, external events that were also chronically repetitive in nature and aggravated existing everyday challenges—bridging current definitions of shocks and stresses.[14] This suggests that the normal pattern of floods in the districts are not a major challenge to the health services. Since floods are projected to gradually become more frequent with climate change, this is an argument for promoting the concept of everyday resilience towards repeated seasonal and occasional floods, that can in turn promote resilience to more acute, extreme shocks.[3,5]

Study limitations

Some desirability bias is likely present among participants at higher levels of authority, such as the health departments, when it was harder to achieve depth and capture informal views than with lower-level participants. Interviews with the participants who were previous students of a data collector were checked for respondent and researcher biases; there were no clear differences. Interviews that gave practical examples of participants' rationale for their actions during floods were considered higher quality in the study. Using a nascent conceptual framework with overlapping dimensions meant data had to be abstracted to a high degree during analysis. We dealt with this by using a data-driven approach during coding and then allowed the categories and themes to be influenced by the framework.

CONCLUSIONS

Public health facilities and health departments appeared to have the capacity to absorb and adapt to seasonal and occasional floods in Cambodia. The boundaries on decisions and actions and pre-existing flood plans for facilities and departments at multiple levels created stability and flexibility when preparing and responding to floods. The apparent success of the system in responding to floods leads us to conclude that strategies that enhance stability and flexibility may foster the capacity for health systems resilience. However, the impact of the floods in this study were minor compared to persistent, system-wide challenges to health service functioning. Health systems that are chronically strained by repeated external

For peer review only - http://bmjopen.bmj.com/site/about/guidelines.xhtml

events may benefit most from health system strengthening efforts to everyday challenges that can pay off during stronger shocks.

For peer review only - http://bmjopen.bmj.com/site/about/guidelines.xhtml

ACKNOWLEDGEMENTS

The authors would like to thank all the clinicians and staff who gave their time to take part in this study. We also thank the data collectors for their outstanding work, Sophea Men for her translation of the transcripts, and Vannarath Te for his contributions during piloting.

FUNDING STATEMENT

This work was supported by the Swedish Research Council (Dnr: 2016-05678)

COMPETING INTERESTS

None declared.

AUTHOR STATEMENT

DDS, PI, CH, HM-A and JVS designed the study. DDS and DT led the data collection. HM-A took part in the piloting. DDS led the data analysis and wrote the manuscript with input from all coauthors. All authors agreed on the final version of the manuscript.

DATA SHARING

No data are available. This is a qualitative study of a small, specific population in two unique geographic regions of rural Cambodia. Making the full data set publicly available could potentially breach the privacy that was promised to participants when they agreed to take part and the ethics approval granted from the National Ethics Committee for Human Research in Cambodia and the Swedish Ethical Review Authority. Therefore, the authors will not make the full transcripts available to a wider audience.

REFERENCES

1 Organization WH. Maintaining essential health services: operational guidance for the COVID-19 context. Geneva, Switzerland: 2020.

2 Buonsenso D, Cinicola B, Kallon MN, *et al.* Child Healthcare and Immunizations in Sub-Saharan Africa During the COVID-19 Pandemic. *Front Pediatr* 2020;**8**. doi:10.3389/fped.2020.00517

3 Kruk ME, Myers M, Varpilah ST, *et al.* What is a resilient health system? Lessons from Ebola. *Lancet* 2015;**385**:1910–2. doi:10.1016/s0140-6736(15)60755-3

4 Haldane V, Ong S-E, Chuah FL-H, *et al.* Health systems resilience: meaningful construct or catchphrase? *The Lancet* 2017;**389**:1513. doi:10.1016/s0140-6736(17)30946-7

For peer review only - http://bmjopen.bmj.com/site/about/guidelines.xhtml

5 Barasa EW, Cloete K, Gilson L. From bouncing back, to nurturing emergence: reframing the concept of resilience in health systems strengthening. Health Policy Plan. 2017;**32**:4.

6 IPCC. Global warming of 1.5°C. An IPCC Special Report on the impacts of global warming of 1.5°C above pre-industrial levels and related global greenhouse gas emission pathways, in the context of strengthening the global response to the threat of climate change, sustainable development, and efforts to eradicate poverty. In Press: 2018.

7 Watts N, Adger WN, Agnolucci P, *et al.* Health and climate change: policy responses to protect public health. *Lancet* 2015;**386**:1861–914. doi:10.1016/s0140-6736(15)60854-6

8 Watts N, Amann M, Ayeb-Karlsson S, *et al.* The Lancet Countdown on health and climate change: from 25 years of inaction to a global transformation for public health. *Lancet Lond Engl* 2018;**391**:581–630. doi:10.1016/S0140-6736(17)32464-9

9 Whitmee S, Haines A, Beyrer C, *et al.* Safeguarding human health in the Anthropocene epoch: report of The Rockefeller Foundation–Lancet Commission on planetary health. *The Lancet* 2015;**386**:1973–2028. doi:10.1016/S0140-6736(15)60901-1

10 Curtis S, Fair A, Wistow J, *et al.* Impact of extreme weather events and climate change for health and social care systems. Env. Health. 2017;**16**.

11 Salas RN, Jha AK. Climate change threatens the achievement of effective universal healthcare. *BMJ* 2019;**366**:l5302. doi:10.1136/bmj.l5302

12 Hanefeld J, Mayhew S, Legido-Quigley H, *et al.* Towards an understanding of resilience: responding to health systems shocks. *Health Policy Plan* 2018;**33**:355–67. doi:10.1093/heapol/czx183

13 Barasa E, Mbau R, Gilson L. What Is Resilience and How Can It Be Nurtured? A Systematic Review of Empirical Literature on Organizational Resilience. Int. J. Health Policy Manag. 2018;**7**:491–503. doi:10.15171/ijhpm.2018.06

14 Turenne CP, Gautier L, Degroote S, *et al.* Conceptual analysis of health systems resilience: A scoping review. [Review]. *Soc Sci Med* 2019;**1**:168–80. doi:10.1016/j.socscimed.2019.04.020

15 Bene C, Wood RG, Newsham A, *et al.* IDS Working Paper 405. Resilience: New Utopia or New Tyranny? Reflection about the potentials and limits of the concept of resilience in relation to vulnerability reduction programmes. Institute of Development Studies 2012.

16 Naimoli JF, Saxena S. Realizing their potential to become learning organizations to foster health system resilience: opportunities and challenges for health ministries in low- and middle-income countries. *Health Policy Plan* 2018;**33**:1083–95. doi:10.1093/heapol/czy100

17 Ministry of Health. National Strategic Plan on Disaster Risk Management for Health, 2015-2019. 2015.

18 National Committee for Disaster Management, Ministry of Planning. Strategic National Action Plan for Disaster Risk Reduction: 2008-2013. Cambodia: 2008.

19 World Food Programme. 2012 Cambodia post-flood relief and recovery survey. Phnom Penh, Cambodia: : WFP 2012.

For peer review only - http://bmjopen.bmj.com/site/about/guidelines.xhtml

20 Ministry of Health Cambodia. Climate Change Strategic Plan for Public Health: Towards increased climate resilience for better health and well-being of all Cambodians. Phnom Penh, Cambodia: 2013.

21 Annear PL, Grundy J, Ir P, *et al.* The Kingdom of Cambodia Health System Review. Asia Pacific Observatory on Public Health Systems and Policies 2015.

22 Sochas L, Channon AA, Nam S. Counting indirect crisis-related deaths in the context of a low-resilience health system: the case of maternal and neonatal health during the Ebola epidemic in Sierra Leone. *Health Policy Plan* 2017;**32**:iii32–9. doi:10.1093/heapol/czx108

23 Kruk ME, Kujawski S, Moyer CA, *et al.* Next generation maternal health: external shocks and health-system innovations. *Lancet* 2016;**388**:2296–306.

24 Blanchet K, Nam SL, Ramalingam B, *et al.* Governance and capacity to manage resilience of health systems: Towards a new conceptual framework. Int J Health Policy Manag. 2017;**6**:5.

25 Thomas S, Keegan C, Barry S, *et al.* A framework for assessing health system resilience in an economic crisis: Ireland as a test case. *BMC Health Serv Res* 2013;**13**:450. doi:10.1186/1472-6963-13-450

26 OECD. Guidelines for resilience systems analysis. 2014.

27 World Bank. World DataBank. 2018.http://databank.worldbank.org/data/home.aspx

28 Royal Government of Cambodia. National Action Plan for Disaster Risk Reduction 2014-2018. Phnom Penh, Cambodia: : Royal Government of Cambodia 2015.

29 Ministry of Health. National guidelines on Complementary Package of Activities for referral hospital development from 2006 to 2010. Cambodia: : Ministry of Health 2006.

30 Bank W. Trends in maternal mortality 2000 to 2017 : Estimates by WHO, UNICEF, UNFPA, World Bank Group and the United Nations Population Division (Vol. 2). Washington, D.C.: : World Bank Group 2019.

31 National Institute of Statistics, Directorate General for Health, ICF International. Cambodia Demographic and Health Survey 2014. Phnom Penh, Cambodia and Rockville, Maryland, USA: : National Institutet of Statistics, Directorate General for Health, and ICF International 2015.

32 National Institute of Statistics, Directorate General for Health, ORC Macro. Cambodia Demographic and Health Survey 2000. Calverton, Maryland: : National Institute of Statistics, Directorate General for Health, ORC Macro 2001.

33 Saulnier DD, Hanson C, Ir P, *et al.* The Effect of Seasonal Floods on Health: Analysis of Six Years of National Health Data and Flood Maps. *Int J Environ Res Public Health* 2018;**15**:665. doi:10.3390/ijerph15040665

34 Malterud K, Siersma VD, Guassora AD. Sample Size in Qualitative Interview Studies: Guided by Information Power. *Qual Health Res* 2016;**26**:1753–60. doi:10.1177/1049732315617444

35 Braun V, Clarke V. Using thematic analysis in psychology. Qual. Res. Psychol. 2006;**3**:77–101.

For peer review only

36 Witter S, Wurie H, Chandiwana P, *et al.* How do health workers experience and cope with shocks? Learning from four fragile and conflict-affected health systems in Uganda, Sierra Leone, Zimbabwe and Cambodia. *Health Policy Plan* 2017;**32**:iii3–13. doi:10.1093/heapol/czx112

37 Gilson L, Barasa E, Nxumalo N, *et al.* Everyday resilience in district health systems: emerging insights from the front lines in Kenya and South Africa. *BMJ Glob Health* 2017;**2**:e000224. doi:10.1136/bmjgh-2016-000224

38 Alonso-Garbayo A, Raven J, Theobald S, *et al.* Decision space for health workforce management in decentralized settings: a case study in Uganda. Health Policy Plan. 2017;**32**:iii59–66. doi:10.1093/heapol/czx116

39 Kelsall T, Heng S. Inclusive healthcare and the political settlement in Cambodia. *New Polit Econ* 2016;**21**:238–55.

40 Liverani M, Chheng K, Parkhurst J. The making of evidence-informed health policy in Cambodia: knowledge, institutions and processes. *BMJ Glob Health* 2018;**3**:e000652. doi:10.1136/bmjgh-2017-000652

41 Saulnier DD, Hean H, Thol D, *et al.* Staying afloat: community perspectives on health system resilience in the management of pregnancy and childbirth care during floods in Cambodia. *BMJ Glob Health* 2020;**5**. doi:10.1136/bmjgh-2019-002272

42 Kittelsen SK, Keating VC. Rational trust in resilient health systems. *Health Policy Plan* 2019;**34**:553–7. doi:10.1093/heapol/czz066

43 Kruk ME, Ling EJ, Bitton A, *et al.* Building resilient health systems: a proposal for a resilience index. *BMJ* 2017;**357**:j2323. doi:10.1136/bmj.j2323

44 RESYST. Using Intersectionality to better understand health system resilience. London, UK: : London School of Hygiene and Tropical Medicine 2017.

For peer review only - http://bmjopen.bmj.com/site/about/guidelines.xhtml

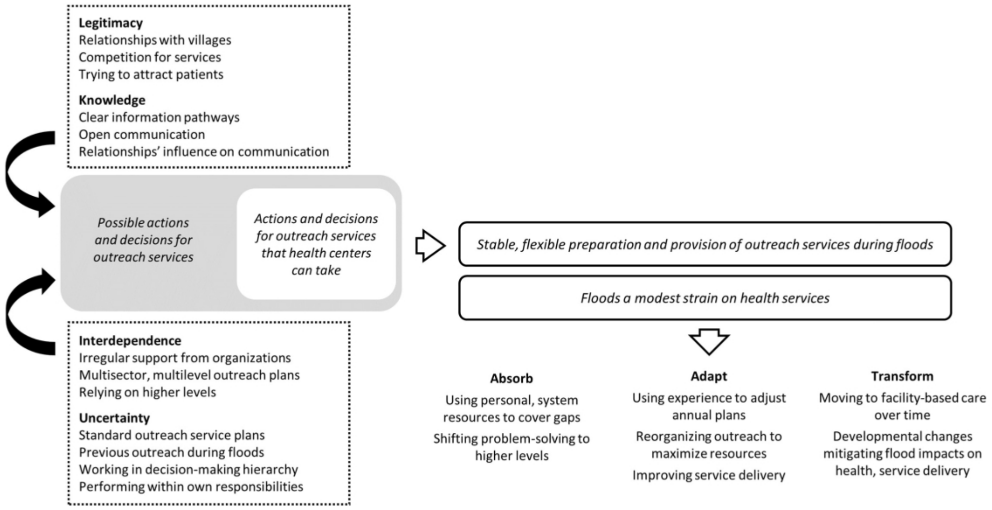

Figure 1. A visual representation of the four dimensions of resilience and the system's capacity to absorb, adapt, and transform as they relate to the main theme, using health center outreach services for antenatal and childbirth care during a flood as an example

196x104mm (300 x 300 DPI)

Supplement 1. Interview guide in English for semi-structured interviews with health facility providers or health department staff.

Flood experiences

Give example of recent flood in the area and confirm event (when, where, length of flood). Can you tell me about the last flood that happened near your [facility/catchment area]?

Explore: Effect on villages and health

Can you describe your experiences of working during a flood?

Explore: Changes/differences in care compared to no floods

Provision and maintenance of services

When there is a flood, what happens to prenatal care services at your [facility/catchment area]? What happens to delivery care services?

Explore: Demand and access to care, staff changes, supplies, medicines, user fees, budget, management from upper levels

Can you describe a time when a woman was not able to get prenatal care at a [facility] during a flood? What happened? What about a time when a woman was not able to get delivery care at a [facility]? What happened then?

Anticipating and coping with uncertainty

How do you prepare prenatal services for the rainy season? How do you prepare delivery services?

Can you share specific examples of things that you do at the [facility/health department] to make sure that pregnant women are able to continue getting care during floods?

External factors influencing the health system

What are some reasons that your [facility/health department] might not be able to provide prenatal care during floods? What about for delivery care?

Explore: Support and work with other departments/sectors/NGOs/committees, transport and access to facilities, supply chain, available funds, staff personal lives and priorities, changes in health, emergencies

What do you think influences pregnant women to come to your facility for prenatal care during floods? What about for delivery care?

Interaction with the community

How does the [facility/health department] work with pregnant women in the village during floods?

Explore: Reasons for visiting other providers or home delivery, outreach in villages, input and accountability with community

In your opinion, how do you think the pregnant women feel about the prenatal care that they can get at [facilities] during floods? What about delivery care?

Explore: Trust and quality, social media, feeling of ownership

Gathering and using knowledge

What kind of decisions do you have to make about services for prenatal care during floods? For delivery care?

Explore: Referring patients to hospital, emergency obstetric cases, sources of information, communication with other departments/facilities/committees, flexibility and ability to make decisions

From your experience working during floods, what have you learned about providing prenatal care during floods? What have you learned about providing delivery care?

Can you give me an example of something you would like to know when there is a flood that would help make prenatal care better during floods? And for delivery care?

For peer review only

COREQ (COnsolidated criteria for REporting Qualitative research) Checklist

A checklist of items that should be included in reports of qualitative research. You must report the page number in your manuscript where you consider each of the items listed in this checklist. If you have not included this information, either revise your manuscript accordingly before submitting or note N/A.

Topic	Item No.	Guide Questions/Description	Reported on Page No.
**Domain 1: Research team and reflexivity**			
Personal characteristics			
Interviewer/facilitator	1	Which author/s conducted the interview or focus group?	6
Credentials	2	What were the researcher's credentials? E.g. PhD, MD	6
Occupation	3	What was their occupation at the time of the study?	6
Gender	4	Was the researcher male or female?	6
Experience and training	5	What experience or training did the researcher have?	6
Relationship with participants			
Relationship established	6	Was a relationship established prior to study commencement?	6
Participant knowledge of the interviewer	7	What did the participants know about the researcher? e.g. personal goals, reasons for doing the research	6
Interviewer characteristics	8	What characteristics were reported about the inter viewer/facilitator? e.g. Bias, assumptions, reasons and interests in the research topic	6
**Domain 2: Study design**			
Theoretical framework			
Methodological orientation and Theory	9	What methodological orientation was stated to underpin the study? e.g. grounded theory, discourse analysis, ethnography, phenomenology, content analysis	6
Participant selection			
Sampling	10	How were participants selected? e.g. purposive, convenience, consecutive, snowball	6
Method of approach	11	How were participants approached? e.g. face-to-face, telephone, mail, email	6
Sample size	12	How many participants were in the study?	6
Non-participation	13	How many people refused to participate or dropped out? Reasons?	7
Setting			
Setting of data collection	14	Where was the data collected? e.g. home, clinic, workplace	6
Presence of non-participants	15	Was anyone else present besides the participants and researchers?	6
Description of sample	16	What are the important characteristics of the sample? e.g. demographic data, date	6
Data collection			
Interview guide	17	Were questions, prompts, guides provided by the authors? Was it pilot tested?	6
Repeat interviews	18	Were repeat inter views carried out? If yes, how many?	6
Audio/visual recording	19	Did the research use audio or visual recording to collect the data?	6
Field notes	20	Were field notes made during and/or after the inter view or focus group?	6
Duration	21	What was the duration of the inter views or focus group?	6
Data saturation	22	Was data saturation discussed?	6
Transcripts returned	23	Were transcripts returned to participants for comment and/or	6

For peer review only

Topic	Item No.	Guide Questions/Description	Reported on Page No.
		correction?	
**Domain 3: analysis and findings**			
Data analysis			
Number of data coders	24	How many data coders coded the data?	7
Description of the coding tree	25	Did authors provide a description of the coding tree?	7
Derivation of themes	26	Were themes identified in advance or derived from the data?	7
Software	27	What software, if applicable, was used to manage the data?	7
Participant checking	28	Did participants provide feedback on the findings?	7
Reporting			
Quotations presented	29	Were participant quotations presented to illustrate the themes/findings? Was each quotation identified? e.g. participant number	7-12
Data and findings consistent	30	Was there consistency between the data presented and the findings?	7-12
Clarity of major themes	31	Were major themes clearly presented in the findings?	7-12
Clarity of minor themes	32	Is there a description of diverse cases or discussion of minor themes?	7-12

Developed from: Tong A, Sainsbury P, Craig J. Consolidated criteria for reporting qualitative research (COREQ): a 32-item checklist for interviews and focus groups. *International Journal for Quality in Health Care*. 2007. Volume 19, Number 6: pp. 349 – 357

Once you have completed this checklist, please save a copy and upload it as part of your submission. DO NOT include this checklist as part of the main manuscript document. It must be uploaded as a separate file.

For peer review only
